# Climate change beliefs, emotions and pro-environmental behaviors among adults: The role of core personality traits and the time perspective

**DOI:** 10.1371/journal.pone.0300246

**Published:** 2024-04-10

**Authors:** Kinga Tucholska, Bożena Gulla, Agnieszka Ziernicka-Wojtaszek

**Affiliations:** 1 Institute of Applied Psychology, Jagiellonian University, Krakow, Poland; 2 Department of Ecology, Climatology and Air Protection, University of Agriculture, Krakow, Poland; Purdue University, UNITED STATES

## Abstract

Climate change and its consequences are recognized as one of the most important challenges to the functioning of the Earth’s ecosystem and humanity. However, the response to the threat posed by the climate crisis still seems inadequate. The question of which psychological factors cause people to engage (or not) in pro-environmental behavior remains without a comprehensive answer. The aim of this study is to establish the links between the cognitive (level of knowledge about climate change and degree of belief in climate myths), emotional (various climate emotions, especially climate anxiety) and behavioral aspects of attitudes towards the climate crisis and their determinants in the form of the Big Five personality domains and time perspectives. The stated hypotheses were verified by analyzing data collected in an online survey of 333 adults using knowledge tests and self-report methods, including psychological questionnaires (*Climate Change Anxiety Scale* by Clayton and Karazsia, *Big Five Inventory–short version* by Schupp and Gerlitz, and *Zimbardo Time Perspective Inventory* by Zimbardo and Boyd), and measurement scales developed for this study (*Climate myth belief scale*, *Climate emotion scale*, and *Inventories of current and planned pro-environmental activities*). The results of stepwise regression analysis demonstrate the importance of the core personality traits and the dominant temporal perspective as determinants of belief in climate change myths, climate anxiety, as well as actual and planned pro-environmental behavior.

## Introduction

According to the latest information from the World Meteorological Organization (2022) [1], the climate situation is dramatically worsening. The last decade was one of the warmest in human history. The anthropogenic nature of climate change is undeniable. However, it is also clear that through policy decisions and systemic solutions aimed at transitioning to a sustainable economy, as well as a range of individual pro-environmental behavior (PEB), people can contribute to slowing or even solving these climate problems.

A number of situational, social, and demographic factors have been identified that influence the extent to which individuals take action to improve the global environmental and climate situation. Research findings indicate that views on climate change and the willingness to address it are dependent on financial resources [2], cultural orientation [3], social influence [4], political views [5], and levels of trust in scientists and authorities [6]. However, some people–despite having the knowledge, resources, role models, social support, and intention to change their behavior to be more pro-environmental–do not engage sufficiently, consistently or systematically in activities that could particularly benefit the environment. This phenomenon is described by environmental and conservation psychologists as an “attitude-behavior gap” [2] or a “concern-behavior gap” [3]. To understand inconsistencies in the PEB domain, it appears crucial to also look for their correlates in knowledge and emotions related to climate change, as well as determinants in personality structures, which explain relatively persistent tendencies to respond in a certain way to situational challenges, irrespective of external factors. Our project is part of a stream of research attempting to describe and understand the “pro-environmental individual” (PEI) [7] facing the climate crisis. Our goal is to fill the knowledge gap on personality traits and temporal orientations as determinants of attitudes toward climate change, a key aspect of which is the willingness to take pro-environmental action, beliefs about climate change and experienced climate emotions. The research model we have adopted also allows us to identify some barriers to engaging in pro-environmental activity that are related to personality traits and time perspective (TP), so this model will also help explain why some people do not engage in pro-environmental activity.

### Mental-emotional responses to and attitudes toward the climate situation

Although the scientific consensus that climate change is real, man-made, and one of humanity’s greatest challenges that must be confronted immediately [8] is now widely known, certain social groups do not share this view. Survey results indicate that only 62% of Americans recognize that global warming is caused by humanity [5]; in the Polish population, this belief is shared by 68.5% of respondents [9]. Deficiencies in knowledge about the climate situation, ways of improving it, and low levels of trust in science are a few of the many possible explanations for unwillingness to engage in PEB. Convictions about the uncertainty of climate change, its distant time horizon, or its natural (but not necessarily anthropogenic) causes, as well as doubts as to its negative consequences, allow the status quo to persist. The lower an individual’s degree of cognitive openness and the greater their rigidity and tendency towards stereotypical thinking, the more easily they succumb to climate myths [10]. However, cognitive factors are not of primary importance. A classic meta-analysis of 128 studies on the correlations between indicators of knowledge and attitudes, attitudes and intentions, and intentions and environmentally responsible behavior indicates that they are weak [11].

Another factor that may prevent people from making behavioral changes to protect the planet’s resources is overwhelming unpleasant emotions brought on by realization of the seriousness of the climate situation. These can range from climate anxiety [12], depression, sadness or apathy [13], to climate despair [14], anger or rage [13]. They may also experience emotions related to self-awareness, such as feelings of shame or guilt, or emotions that do not involve discomfort, such as indifference, active hope [15] or compassion [16]. Many people take small steps that they believe can stop climate change. However, these require self-sacrifice, as giving up a comfortable lifestyle based on unlimited consumption entails effort and economic costs, and therefore the changes made are often unsystematic. Even individuals who are strongly committed to climate activism become progressively burned out as they cannot see the impact of the beneficial changes they make [17].

Pro-environmental attitudes (PEAs) depend on both knowledge about the climate situation and beliefs and convictions about its anthropogenic origin. The following types of climate attitudes can be distinguished [18, 19]: lack of interest; belittling the problem; active denial of the problem (climate denialism); rigidity (no denial of the problem but a desire to maintain one’s current lifestyle in spite of it); dramatization, which reduces motivation and energy for action; active preparation for an apocalypse in any form (represented by so-called “preppers“); and realism and commitment associated with climate-related emotions which are appropriate to the situation and taking steps to contribute to improving the situation.

In the view of Aronson, Wilson and Akert [20], the classics of social psychology attitudes are a combination of a cognitive, emotional and behavioral component. The cognitive component of an attitude is the belief or idea associated with a particular object. The affective component includes the individual’s evaluation and valuing of the object, as well as the emotions associated with it. The behavioral component includes an action or predisposition to act directed towards that object. However, declared attitudes tend to be a relatively poor predictor of behavioral action, as both attitudes and behavior are influenced by many other factors (personality factors, situational factors and, among these, especially social factors). Consequently, it is more accurate to predict behavior on the basis of attitudes with consideration of action in the long term [21]. However, it is also worth examining attitudes that are more specific, towards a well-defined object, as such attitudes have greater predictive value. The higher the accuracy of the diagnosis of attitudes in the three aspects of their manifestation (cognitive, emotional and behavioral), the stronger the link between attitudes and action itself [22].

### Core dimensions of personality, the time perspective, and environmentalism

Personality is defined as the characteristic, relatively stable pattern of thoughts, feelings, and behaviors exhibited by individuals. As the source of beliefs, values, and motives, it can be considered a fundamental explanation for individual differences in PEA and PEB. Initially, psychologists studying the personality correlates of PEA and PEB focused on specific narrow aspects and personality traits. This is reflected in work by Hines, Hungerford and Tomera [23] that analyzed and summarized the initial findings of research on responsible environmental behavior. This first meta-analysis confirmed that statistically significant correlates of PEB are PEA (r = 0.35), locus of control (r = 0.37), personal responsibility (r = 0.33), economic orientation (r = 0.16), and verbal commitment (r = 0.49).

A recent meta-analysis of data from 38 sources, collected from nearly 50,000 individuals [24], verified the associations between the core personality dimensions described in the Big Five model [25] and the six-factor HEXACO model [26], PEA, and PEB. Five-Factor Theory of Personality includes five dimensions. These are [27]: (1) Neuroticism (characteristic adaptation: low self-esteem, irrational perfectionistic beliefs, pessimistic attitudes), (2) Extraversion (characteristic adaptation: social skills, numerous friendships, enterprising vocational interests, participation in sports, club memberships), (3) Openness to Experience (characteristic adaptation: interest in travel, many different hobbies, knowledge of foreign cuisine, diverse vocational interests, friends who share tastes), (4) Agreeableness (characteristic adaptation: compliance, forgiving attitudes, belief in cooperation, inoffensive language, reputation as a pushover), (5) Conscientiousness (characteristic adaptation: achievement striving, leadership skills, long-term plans, organized support network, technical expertise). In addition to the above-mentioned five factors, the HEXACO model includes sixth dimension which is Honesty-Humility, a basic personality trait representing “the tendency to be fair and genuine in dealing with others, in the sense of cooperation with others even when one might exploit others without suffering retaliation” [26].

The highest correlation coefficients were found between openness, honesty-humility, and PEAi (r = 0.22 and 0.20, respectively), as well as with PEB (r = 0.21 and 0.25, respectively). Agreeableness, conscientiousness, and, to a slightly lesser extent, extraversion were also found to be related to PEA (r = 0.15, 0.12, and 0.09) and PEB (r = 0.10, 0.11, and 0.10). A later study by Soutter and Mõttus [28] essentially confirmed these relationships. Openness appears to correlate most strongly with PEA and PEB (r = 0.46 and 0.35), followed by agreeableness (r = 0.34 and 0.25, respectively) and conscientiousness (r = 0.16 and 0.18). Extraversion and neuroticism did not show statistically significant associations with PEA, but the facet-level traits expressing them appeared to be related to PEB (r = 0.12 and -0.09).

Among the basic personality traits, openness appears to be the most important in the context of relationships with the environment [24]. Openness is associated with the capacity for flexible, abstract thinking, which is necessary for the individual to imagine the complex temporal and spatial consequences of the climate crisis. Openness may also influence pro-environmental behavior indirectly, through values held [29]. Political orientation was found to be strongly associated with PEA [6], and openness to experience may be partly responsible for this effect.

As Pahl and colleges stated [30] most basic temporal dimension of climate change is its extension into the future. While impacts are already happening, the most significant and far-reaching impacts of climate change lie in the future. A further temporal aspect of climate change which complicates temporal distance is the time lag between cause and effect. The climate effects we see now are related to carbon emissions that entered the atmosphere a long time ago. Even if we stopped all additional carbon emissions today, the carbon already in the atmosphere will continue to have impacts for centuries. Environmental engagement evokes two types of dilemmas: social (individual interest vs. collective interests) and temporal concern (short- vs. long-term interests, immediate vs. delayed consequences of one’s actions) [31]. The psychological variable that helps to explain individual differences in decision-making while taking into account time frames is time perspective (TP). According to Zimbardo and Boyd [32], TP is “the often non-conscious personal attitude that each of us holds toward time and the process whereby the continual flow of existence is bundled into time categories that help to give order, coherence, and meaning to our lives” (p. 51). People can be distinguished by their preferred time perspective. Some people habitually prefer a future time perspective, characterized by subjectively important and meaningful mental representations of the future and a focus on future goals and achievements, whereas others habitually think more about the past or the present.

Measurement of TP involves the assessment of relative preferences for past/present/future events, experiences and goals, and it helps to understand engagement or apathy towards environmental problems. Research on environmental engagement and TP is most commonly conducted with the *Consideration of Future Consequences Scale* [33] which captures the tendency to focus on the distant future or the immediate consequences of one’s actions. Research is also conducted using the *Zimbardo Time Perspective Inventory* [34], which measures five various time frames (past positive and negative, present hedonistic and fatalistic, and future).

A meta-analysis of studies conducted by Milfont, Wilson, and Diniz [35], using data from 19 independent samples and 6,301 respondents from seven countries, indicates that the future TP has a more significant relationship with PEBs (medium effect; r(k = 13) = 0. 26) and PEAs (small effect; r(k = 10) = 0.17) than the combined score of the past-present perspective (significant but trivial effect found only for PEBs; r(k = 4) = 0.06)). However, these results may be due to the fact that far fewer studies included a full temporal perspective (not only on the future, but also on the present and past). Additionally, it is important to note that the association between the future perspective and PEB is stronger than its association with PEA.

There is a lack of research considering the combined effects of basic personality traits and TP on PEB; our study is designed to fill this gap.

### Goal, research model and hypotheses

**Fig 1:**
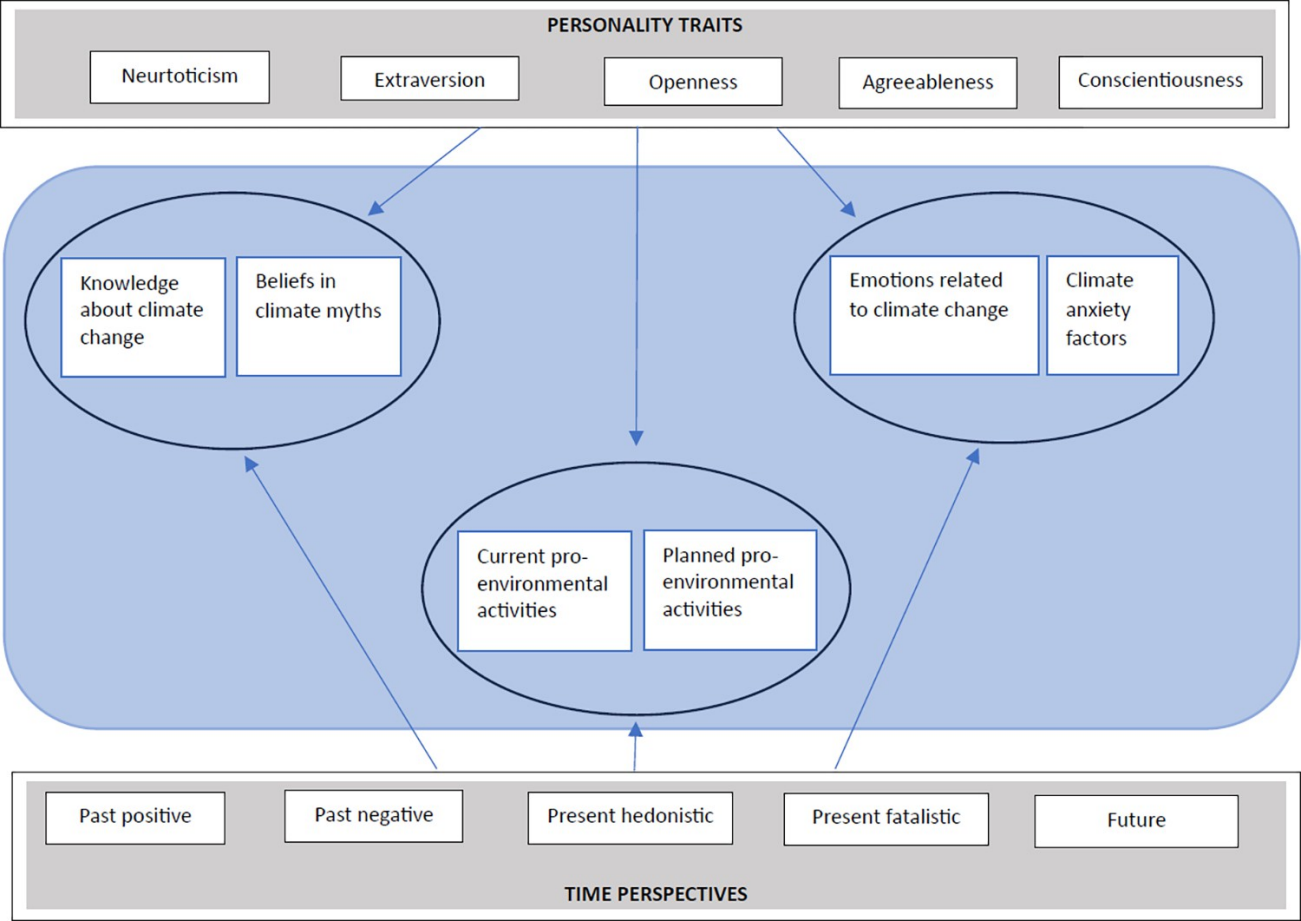
Research model.

Accordingly, in line with the three-component concept of attitudes [20] we consider three components of attitudes toward climate change: the cognitive component (knowledge, beliefs, and misconceptions about the climate and its changes), the affective component (emotional reactions in the face of climate change), and the behavioral component (willingness to act and how to behave in relation to progressive climate change). The research goal was to determine the level of knowledge about climate change and the degree of belief in climate myths in a sample of adult Poles, the emotional reactions aroused in response to the climate-environmental situation, and forms and frequency of PEB undertaken and planned by the subjects. We also aimed to explore the interplay between these three aspects of attitudes to climate change. The main objective of the study was to capture the intrapsychic variables (basic personality traits and TP) that are relevant to explaining the above three aspects of attitudes to climate change. Based on the information presented above, the following general research model (see Fig 1) and hypotheses were made:

H1a. Openness predict the level of knowledge about climate changes—the higher the level of openness the higher the level of knowledge about climate change.
H1b. Conscientiousness predict the level of knowledge about climate changes—the higher the level consciousness, the higher the level of knowledge about climate change.
H1c. Future time perspective predict the level of knowledge about climate changes—the higher the level of future time perspective, the higher the level of knowledge about climate change.
H2a. Openness predicts the level of belief in climate myths—the lower the level of openness, the higher the level of belief in climate myths.
H2b. Neuroticism predicts belief in climate myths—the lower the level of neuroticism, the higher the level of belief in climate myths.
H2c. Past positive perspective predicts belief in climate myths–the higher the level of past positive perspective, the higher the level of belief in climate myths.
H3a. Neuroticism predicts the level of cognitive and emotional impairment as aspects of climate anxiety–the higher the level of neuroticism, the higher the level of cognitive and emotional impairment.
H3b. Conscientiousness predicts the level of functional impairment as aspect of climate anxiety–the lower the level of conscientiousness, the higher the level of functional impairment.
H3c. Future time perspective predicts the experience of climate change–the higher the level of future time perspective, the higher the level of climate change experience.
H4a. Openness predicts behavioral engagement in pro-environmental activity–the higher the level of openness, the higher the level of behavioral engagement.
H4b. Conscientiousness predicts current pro-environmental activity–the higher the level of conscientiousness, the higher the level of current pro-environmental activity.
H4c. Present hedonism predicts current pro-environmental activity–the lower the level of present hedonism, the higher the level of current pro-environmental activity.
H4d. Present fatalism predicts current pro-environmental activity–the lower the level of present fatalism, the higher the level of current pro-environmental activity.
H4e. Future time perspective predicts current pro-environmental activity–the higher the level of future time perspective, the higher the level of current pro-environmental activity.
H5a. Openness predicts planned pro-environmental activity–the higher the level of openness, the higher the level of planned pro-environmental activity.
H5b. Neuroticism predicts planned pro-environmental activity–the higher the level of neuroticism, the higher the level of planned pro-environmental activity.
H5c. Future time perspective predicts planned pro-environmental activity–the higher the level of future time perspective, the higher the level of planned pro-environmental activity.

## Materials and methods

### Participants

A total of 333 participants, adult Poles, took part in the study (30.33% male), with a mean age of 29.14 years (SD = 11.20, range 18–80); see Table 1 for detailed participant characteristics.

**Table 1:** Sample characteristics.

		N	Percent
**Gender**			
	Male	101	30.33%
	Female	229	68.77%
	Non-binary	3	0.90%
**Residence**			
	Big city	200	60.06%
	Medium/small town	69	20.72%
	Village	64	19.22%
**Education**			
	Primary	5	1.51%
	Secondary	141	42.47%
	Higher	186	56.02%
**Offspring**			
	Yes	67	20.12%
	No	266	29.88%

*Note*. Due to missing data the total *N* does not always add up to 333 for each.

### Measures

*Climate myth belief scale*–a method developed for this project (see S1 Appendix). A person assigns beliefs (representing so-called climate myths, e.g., “Humans are too insignificant to influence the planet’s climate”) a rating from 1 to 5 stars; the more stars the participant chooses, the stronger his or her belief that a given statement is true. One star (coded as 1 point) means that the respondent does not agree with the statement. The score is the sum of the points (min 10; max 50).

*Knowledge test about climate and its changes*–a method developed for the purposes of this project (see S2 Appendix). It consists of 15 questions measuring the participant’s level of knowledge about the causes of climate change, its consequences, the anthropogenic sources of the crisis, and possible ways to improve the environment. It is a multiple-choice test: four answer options are given, including one correct answer (scored). The knowledge indicator is the sum of the points in the test (min. 0; max. 15).

*Climate Change Anxiety Scale* (CCA) by Clayton and Karazsia [36] is 22-item scale with a 5-point Likert-type response format for assessing climate anxiety as a psychological response to climate change. Scores on four factor scales are assigned: (1) cognitive-emotional impairment—reflected in rumination, difficulty sleeping or concentrating, and nightmares or crying; (2) functional impairment—high ratings on this factor indicate that concern about climate change is interfering with a person’s ability to work or socialize; (3) experience of climate change; and (4) behavioral engagement—not just engaging in sustainable behavior, but endorsing the significance of a behavioral response. Scales 1–3 measure the severity of climate change anxiety; scale 4 measures pro-climate engagement.

*Climate emotion scale* (CES)–a method developed for this project (see S3 Appendix) based on *Climate Change Distress* by Searle and Gow [37], to which four items were added (”mobilized”, “indifferent”, “full of energy”, and “calm”). The subject’s task is to complete the sentence “Thinking about climate change right now makes me feel…” by defining the intensity of 15 emotions and feelings, assigning 1–5 points to each one. A score of 1 means that the feeling is minimal or absent. Indices are established for each of the 15 climate emotions (min. 1; max. 5).

*Inventory of current pro-environmental activities*–a method developed for this project (see S4 Appendix). It is a 10-item list of various actions that can mitigate the effects of climate change (e.g., saving electricity or water, or reducing travel). The participant indicates the extent to which they currently carry out a given action or activity using a 5-point scale, where 1 = not at all, 2 = rarely, 3 = quite often, 4 = often, 5 = almost always. The index of frequency of current pro-environmental activities is the sum of the scores.

*Inventory of planned pro-environmental activities*–a method developed for this project. It is a 10-item list of actions that can mitigate the effects of climate change. The respondent indicates the extent to which they want and intend to implement these behaviors in the future by rating each on a 5-point scale, where 1 = not at all, 2 = rarely, 3 = quite often, 4 = often, 5 = almost always. A summary index of the frequency of planned pro-environmental behaviors is established.

*Big Five Inventory–short version* (BFI-S) by Schupp and Gerlitz [38], adapted by Strus, Cieciuch, and Rowinski. This is a shortened, 15-item version of a questionnaire for measuring the five basic dimensions of personality. The subject responds to 15 statements using a graphically represented 7-point scale whose anchors are described as 1 = strongly NO, 7 = strongly YES. Scores are assigned on five factor scales: neuroticism, extraversion, openness, agreeableness, and conscientiousness.

*Zimbardo Time Perspective Inventory* (ZTPI) by Zimbardo and Boyd [34], 15-item version in Polish translation developed by Cybis, Rowinski, Przepiórka, and Meisner. An inventory for measuring TP, i.e., how one relates to the past, present, and future. The respondent indicates how well each of the 15 statements characterizes them using a 5-point scale: 1 = completely disagree, 2 = mostly disagree, 3 = hard to say, 4 = mostly agree, 5 = completely agree. Factor scales are present hedonistic, present fatalistic, past positive, past negative, and future.

### Procedure

The self-report study was conducted online. Participants were recruited form among students of Jagiellonian University in Krakow and the Agricultural University in Cracow. The “snowball sampling” method was then used to extend the study population to include non-students. An invitation to participate in the study was posted on the university’s online research platform, along with a link to the survey form. Everyone who was interested was provided with information about the purpose of the study, the anonymous nature of participation in the study, the use of the results for research purposes only, and the possibility of withdrawing from the study at any stage without any consequences before taking part. Everyone who chose to participate in the study confirmed that he or she was of legal age and gave written informed consent to participate in the study. Respondents were asked to share information about the study and a link to the form with other interested parties outside the student body. The subjects were not compensated for their participation. The Research Ethics Committee at the Institute of Applied Psychology at Jagiellonian University in Krakow gave a positive opinion of the project.

## Results

A multiple testing and comparisons between variables including Pearson’s correlation (as a form of preliminary analysis) and a full stepwise procedure was applied, that is, a combination of forward and backward analysis. The analyses were done by means of the software PS IMAGO PRO (SPSS 26) [39].

### Preliminary analysis

Descriptive statistics of the indicators along with reliability measures are included in Table 2. Correlation analyses (see Table 3) were conducted as the preliminary analysis using the data obtained in the study.

**Table 2:** Descriptive statistics and reliability of the measures.

*Scale*	Number of items	*M*	*SD*	Cronbach’s *alpha*
*Climate myth belief scale*	10	16.22	6.03	0.83
*Test of knowledge about climate and its changes*	15	11.44	1.59	0.85
*Climate change anxiety scale*				
Cognitive-emotional impairment	08	1.60	0.66	0.84
Functional impairment	05	1.52	0.76	0.85
Experience of climate change	03	2.18	1.06	0.79
Behavioral engagement	06	3.85	0.67	0.70
*Climate emotions scale*				0.92
Sad	01	2.97	1.42	
Depressed	01	2.94	1.43	
Angry	01	2.72	1.45	
Powerless	01	2.94	1.39	
Scared	01	2.71	1.46	
Worried	01	3.20	1.42	
Mobilized	01	2.80	1.23	
Indifferent	01	1.56	1.03	
Anxious	01	2.50	1.33	
Helpless	01	2.54	1.40	
Tense	01	2.11	1.27	
Concerned	01	2.95	1.38	
Hopeless	01	2.16	1.26	
Full of energy	01	1.93	1.12	
Calm	01	1.78	1.14	
*Inventory of current pro-environmental activities*	10	35.20	6.48	0.79
*Inventory of planned pro-environmental activities*	10	38.23	8.06	0.78
*Big Five Inventory–short version*				
Neuroticism	03	4.46	1.30	0.64
Extraversion	03	3.91	1.30	0.66
Openness	03	5.37	1.04	0.64
Agreeableness	03	4.97	1.12	0.52
Conscientiousness	03	5.18	1.04	0.63
*Zimbardo Time Perspective Inventory*				
Present hedonistic	03	4.11	0.77	0.65
Present fatalistic	03	4.75	0.67	0.63
Past positive	03	3.17	0.88	0.61
Past negative	03	3.85	0.62	0.81
Future	03	3.62	0.95	0.79

**Table 3:** Pearson’s correlation matrix of variables in the study.

*Scale*	1	2	3	4	5	6	7	8	9	10	11	12	13	14	15	16	17	18
1. Belief in climate myths	-																	
2. Knowledge about climate and its changes	-,15**	-																
3. Cognitive-emotional impairment	-,27**	.04	-															
4. Functional impairment	-,25**	.08	.72**	-														
5. Experience of climate change	-,19**	.12*	.42**	.43**	-													
6. Behavioral engagement	-,24**	.14*	.41**	.39**	.36**	-												
7. Current pro-environmental activity	-.19**	.22**	.33**	.36**	.28**	.60**	-											
8. Planned pro-environmental activity	-.34**	.13*	.44**	.41**	.31**	.67**	.65**	-										
9. Neuroticism	-,13*	.06	.16**	.11*	.06	.15**	.09	.20**	-									
10. Extraversion	-,06	.08	.02	.02	.02	.08	.13*	.06	.02	-								
11. Openness	-,10	.08	.19**	.12*	.12*	.25**	.37**	.29**	-.03	.27**	-							
12. Agreeableness	,00	-.01	-.01	-.04	-.08	.07	-.06	.05	-.24**	.07	.08	-						
13. Conscientiousness	,10	.04	-.08	-.10	-.04	.10	.15**	.08	-.18**	-.17**	.14**	.16**	-					
14. Present hedonistic	-,03	.03	.14**	.09	.09	.11*	.11*	.15**	-.05	.23**	.13*	.21**	.21**	-				
15. Present fatalistic	,04	.03	.01	-.04	-.03	.13*	.09	.13*	-.25**	.22**	.17**	.72**	.69**	.61**	-			
16. Past positive	,14*	-.06	-.06	-.12*	-.10	.01	-.03	-.01	-.10	.19**	.14*	.30**	.18**	.27**	.37**	-		
17. Past negative	,11*	.01	-.04	-.10	-.06	.15**	.09	.11	-.15**	.19**	.14	.48**	.68**	.43**	.50**	,68**	-	
18. Future	,07	.06	-.02	-.06	-.01	.18**	.14*	.12*	-.02	.04	.02	.15**	.67**	.16**	.79**	.14*	.76**	-

Note

*: *p* <, 05

**: *p* <, 01; *N* = 333

#### Knowledge about the climate crisis and their correlates

Analysis of the knowledge test results showed that the respondents had fairly reliable knowledge about the anthropogenic causes of climate change (96.7% correct answers), recognition of carbon dioxide as a gas causing the greenhouse effect (94.89%), observed extreme weather events (96.1%), knowledge about renewable energy (98.2%), and the need to take adaptive actions in response to climate change (91.6%). On the other hand, respondents were least responsive to questions related to the natural causes of climate change (56.76%), sources of methane (55.26%), the onset of rising temperatures (53.75%), changes in the nature of precipitation in Poland (55.26%), and knowledge of international organizations working on climate issues (53.75%). The lowest results were recorded in response to the question about the rate of temperature increase in Poland (33.93%). Belief in climate myths was low in the surveyed population. The myths that persisted among respondents included the belief that the increase in average global temperatures is due to natural causes (M = 2.10, SD = 1.08); that the climate has changed before, and therefore the current changes are nothing special (M = 2.23, SD = 1.19); and the belief that the average citizen has no influence on climate policy (M = 2.01, SD = 1.20).

The correlation between the indices of climate knowledge and belief in climate myths is negative but weak (r = -0.15; p = 0.008). The higher the index of climate knowledge, the higher the index of pro-environmental actions currently taken and planned for the future. This relationship is weak but significant at r = 0.22 (p < 0.01) for current and r = 0.13 (p < 0.01) for planned PEBs. The higher the level of belief in climate myths, the lower the pro-climate activity currently undertaken (r = -0.19, p < 0.01) and planned for the future (r = -0.34, p < 0.01).

#### Emotions related to climate change and their correlates

The most frequently expressed emotions in the face of the climate change situation (see Table 2) are worry, sadness, concern, depression, anger, and helplessness. The least frequent were calmness and indifference. The greater the belief in climate myths, the less intense the negative emotions (depressed, sad, scared, concerned, worried, angry, tense, anxious, powerless, and hopeless; r = -0.36 to r = -0.16), and the stronger the feelings of indifference (r = 0.36), calmness (r = 0.37), and energy (r = 0.14). The climate knowledge index does not correlate with any of the climate emotions.

Neuroticism correlates positively with a range of climate emotions indicative of distress (depressed, sad, angry, powerless, scared, worried, anxious, helpless, tense, concerned, hopeless; r = 0.18 to r = 0.29; p < 0.01) and shows a negative relationship with feelings of indifference (r = - 0.16; p < .01) and calmness (r = -0.29; p < 0.01) toward the climate situation. Extraversion and agreeableness correlate positively but weakly with feelings of mobilization (r = 0.18 and r = 0.16, respectively, p < 0.01) and energy (r = 0.13 and r = 0.16, respectively; p < 0.01). Openness is positively associated with climate change-related emotions of sadness and anger, as well as to feeling depressed, scared, worried, anxious, tense, concerned, hopeless, and mobilized (r = 0.15 to r = 0.23, p < 0.01); it is negatively related to indifference (r = -.019, p < 0.01). Conscientiousness was associated with feeling mobilized, calm, and full of energy, while a negative relationship was shown with feeling scared, helpless, and hopeless.

The statistically significant correlational relationships between TP and climate change-related emotions are weak. The present hedonistic perspective is associated with experiencing the widest range of emotions–feeling sad, depressed, angry, and helpless, but also mobilized–and with a low level of indifference. The present fatalistic perspective is positively correlated with mobilization and negatively correlated with indifference and hopelessness. This may be due to perception of the negative aspects of multiple areas of current reality, which reduces the relative perceived significance of climate issues so that they do not evoke negative emotions. Past positive is associated with indicators of mobilization and energy surge. Past negative is associated with indicators of anxiety and hopelessness, as well as with feelings of mobilization and energy; it is negatively associated with indifference, helplessness, and hopelessness. Future perspective has a positive relationship with feelings of mobilization and energy and a negative relationship with helplessness.

As measured by the CCA scale, climate anxiety indices of cognitive-emotional impairment and functional impairment are positively weakly related to neuroticism and openness. Additionally, openness shows a weak relationship with the index of personal experience of the effects of climate change. Examination of the correlational links between the climate anxiety indices and TP reveals a weak positive association between the present hedonistic perspective and cognitive-emotional impairment; it also reveals a weak negative association between past positive and functional impairment.

#### Current and planned pro-environmental behaviors and their correlates

Positive relationships of weak to moderate strength were found between the index of current PEB and almost all emotions measured by the CES; only indifference correlates negatively with current activity. Helplessness and calmness are two of the fifteen emotional states that show no relationship with the index of current environmental action. The intensity of planned environmental actions correlates significantly with the entire spectrum of climate emotions, indicating both distress and positive feelings (mobilization, energy); only the feelings of indifference and calmness correlate negatively. The correlation coefficients between climate change-related emotions and planned environmental actions are higher than in the case of actions already taken.

The intensity of currently undertaken PEBs is positively related to extraversion and conscientiousness (weak correlation) and openness (moderate correlation). Pro-environmental actions are also positively related to future and present hedonistic TPs (weak correlations). Pro-environmental action planning is related to neuroticism and openness; it is also related to hedonistic and fatalistic present and future TPs (weak correlations).

### Predictors of climate change knowledge and belief in climate myths

Baseline personality traits and TP were not statistically significant predictors of climate knowledge (see Table 4), thus hypotheses H1a, H1b, and H1c must be rejected. The statistically significant predictors of belief in climate myths were: past positive TP, which explained 1.92% of the variance in the dependent variable; openness, which was negative and explained 1.46% of the variance in the dependent variable; and neuroticism, which was negative and explained 1.29% of the variance. Overall, the predictors in the model explained 5% of the variance in the dependent variable; this value was statistically significant (p = 0.002). Hypothesis H2a, which postulated that the lower the level of openness, the higher the level of belief in climate myths, has been positively verified (Beta = -.12; p = .025). Hypothesis H2b, which postulated that the lower the level of neuroticism, the higher the level of belief in climate myths has been confirmed (Beta = -.11; p = .035). Hypothesis H2c, which postulated that the higher the level of past positive perspective, the higher the level of belief in climate myths has been also positively verified (Beta = 14; p = .011).

**Table 4:** Personality domains and time perspective as predictors of belief in climate myths and knowledge about climate change.

Dependent variable	Predictors	*B*	*Beta*	*t*	*p*
Climate myth belief *R* = .21; *R*^*2*^ = .05 *F* = 5.23; *p* = .002	(Constant)	19.32		8.31	< .001
	Past positive	.96	.14	2.57	.011
	Openness	-.71	-.12	-2.25	.025
	Neuroticism	-.53	-.11	-2.11	.035
Knowledge about climate and its changes	No predictors entered the regression model				
*F* = 1.06; *p* = .386					

### Predictors of climate anxiety

The statistically significant predictors of cognitive and emotional impairment (which is one of the factors of climate anxiety) are as follows (see Table 5): openness–positive, explaining 3.64% of the variance in the dependent variable; neuroticism–positive, explaining 2.21% of the variance in the dependent variable; present hedonistic perspective–positive (2.17% of the variance), and conscientiousness–negative (1.24% of the variance). Together, the predictors in the model explained 9% of the variance in the dependent variable; this value was statistically significant (p < 0.001). Hypothesis H3a, which postulated that the lower the level of neuroticism, the higher the level of belief in climate myths, has been positively verified (Beta = .15; p = .005).

**Table 5:** Personality domains and time perspective as predictors of climate anxiety.

Dependent variable	Predictors	*B*	*Beta*	*t*	*p*
*F* = 1.06; *p* = .386					
Cognitive- emotional impairment *R* = .30; *R*^*2*^ = .09 *F* = 8.37; *p* < .001	(Constant)	.44		1.41	.160
	Openness	.12	.19	3.63	< .001
	Neuroticism	.08	.15	2.82	.005
	Present hedonistic	.13	.15	2.80	.005
	Conscientiousness	-.07	-.12	-2.12	.035
Functional impairment *R* = .25; *R*^*2*^ = .06 *F* = 5.48; *p* < .001	(Constant)	1.27		3.94	< .001
	Openness	.11	.15	2.67	.008
	Past positive	-.14	-.16	-2.83	.005
	Present hedonistic	.14	.14	2.52	.012
	Conscientiousness	-.09	-.12	-2.18	.030
Experience of climate change *R* = .20; *R*^*2*^ = .04 *F* = 4.67; *p* = .003	(Constant)	1.43		3.43	.001
	Openness	.12	.12	2.21	.028
	Past positive	-.18	-.15	-2.71	.007
	Present hedonistic	.16	.12	2.12	.035
	Present fatalistic	1.71	.14	2.66	.008

The predictors of functional impairment are: the past positive perspective–negative, explaining 2.28% of the variance in the dependent variable; openness–positive, explaining 2.04% of the variance in the dependent variable; present hedonistic perspective–positive, explaining 1.81% of the variance in the dependent variable; and conscientiousness–negative, explaining 1.36% of the variance. Together, the predictors in the model explained 6% of the variance in the dependent variable; this value was statistically significant (p < 0.001). Hypothesis H3b, which postulated that the lower the level of conscientiousness, the higher the level of functional impairment, has been confirmed (Beta = -.12; p = .03).

The following were revealed as experience of climate anxiety predictors: openness–positive, explaining 1.42% of the variance in the dependent variable; past positive perspective–negative (2.14% of the variance); and present hedonistic perspective–positive (1.30% of the variance). In total, the predictors in the model explained 4% of the variance in the dependent variable; this value was statistically significant (p = 0.003). Hypothesis H3c must be rejected because the results of the study do not indicate that the future time perspective predicts the experience of climate change.

### Predictors of pro-environmental behavior

According to the data presented in Table 6, statistically significant predictors of behavioral engagement (measured by CCA) are openness–positive, explaining 6.44% of the variance in the dependent variable; future TP–positive, explaining 3.05% of the variance; and neuroticism–positive, explaining 2.53% of the variance. In total, the predictors in the model explained 12% of the variance in the dependent variable; this value was statistically significant (p < 0.001). Hypothesis H4a, which postulated that openness predicts behavioral engagement in pro-environmental activity–the higher the level of openness, the higher the level of behavioral engagement, has been confirmed (Beta = .25; p < .001).

**Table 6:** Personality domains and time perspective as predictors of pro-environmental behavior.

Dependent variable	Predictors	*B*	*Beta*	*T*	*p*
Behavioral engagement *R* = .34; *R*^*2*^ = .12 *F* = 14.80; *p* < .001	(Constant)	2.16		8.43	< .001
	Openness	.16	.25	4.90	< .001
	Future	.12	.17	3.38	.001
	Neuroticism	.08	.16	3.08	.002
Current pro-environmental activity *R* = .41; *R*^*2*^ = .17 *F* = 21.95; *p* < .001	(Constant)	22.12		9.29	< .001
	Openness	2.34	.38	7.47	< .001
	Future	1.01	.15	2.90	.004
	Agreeableness	-.63	-.11	-2.14	.033
Planned pro-environmental activity *R* = .38; *R*^*2*^ = .15 *F* = 18.69; *p* < .001	(Constant)	12.10		3.02	.003
	Openness	2.11	.27	5.27	< .001
	Neuroticism	1.50	.24	4.60	< .001
	Present fatalistic	1.71	.14	2.66	.008

The following proved to be statistically significant predictors of current PEB (measured by *Inventory of current pro-environmental activities*): openness–positive, explaining 14.13% of the variance of the dependent variable; future TP–positive, explaining 2.13% of the variance; and agreeableness–negative, explaining 1.16% of the variance. In total, the predictors in the model explained 17% of the variance in the dependent variable; this value was statistically significant (p < 0.001). Hypotheses H4b, H4c, H4d have been rejected. Hypothesis H4e, which postulated that the higher the level of future time perspective, the higher the level of current pro-environmental activity, has been positively verified (Beta = .15; p = .004).

Planned PEB has the following personality predictors: openness–positive, explaining 7.22% of the variance; neuroticism–positive, explaining 5.50% of the variance; and present fatalistic perspective–positive, explaining 1.84% of the variance. Together, the predictors in the model explained 15% of the variance in the dependent variable; this value was statistically significant (p < 0.001). Hypothesis H5a, which postulated that the higher the level of openness, the higher the level of planned pro-environmental activity, has been confirmed (Beta = .27; p < .001) as well as hypothesis H5b, which stated that the higher the level of neuroticism, the higher the level of planned pro-environmental activity (Beta = .24; p < .001). Hypothesis H5c, which postulated that future time perspective predicts planned pro-environmental activity has been rejected.

## Discussion

Although the Big Five personality traits and TPs are relatively well-recognized constructs and are regarded as important for understanding environmental issues, there has been no previous research on their interconnectedness and combined empirical relation to PEB. Confirming the results of previous studies, our findings provide additional empirical evidence that the core personality domains and diverse TPs underlie PEB. Moreover, their associations with belief in climate myths, a broad spectrum of climate emotions, and climate anxiety indicate that both the Big Five traits and TP are important constructs to be considered in psychological studies related to environmental issues.

The complex research model adopted was essentially concerned with the influence of core personality traits and individual TPs on cognitive, emotional and behavioral aspects of attitudes toward climate change. Seventeen partial hypotheses were formulated and tested in the course of the study. A summary of the verification effects is presented in Table 7.

**Table 7:** Summary of the effects of verification of research hypotheses.

Research hypothesis	Effect of verification
H1a. Openness predict the level of knowledge about climate changes—the higher the level of openness the higher the level of knowledge about climate change.	Rejected
H1b. Conscientiousness predict the level of knowledge about climate changes—the higher the level consciousness, the higher the level of knowledge about climate change.	Rejected
H1c. Future time perspective predict the level of knowledge about climate changes—the higher the level of future time perspective, the higher the level of knowledge about climate change.	Rejected
H2a. Openness predicts the level of belief in climate myths—the lower the level of openness, the higher the level of belief in climate myths.	Confirmed
H2b. Neuroticism predicts belief in climate myths—the lower the level of neuroticism, the higher the level of belief in climate myths.	Confirmed
H2c. Past positive perspective predicts belief in climate myths–the higher the level of past positive perspective, the higher the level of belief in climate myths.	Confirmed
H3a. Neuroticism predicts the level of cognitive and emotional impairment as aspects of climate anxiety–the higher the level of neuroticism, the higher the level of cognitive and emotional impairment.	Confirmed
H3b. Conscientiousness predicts the level of functional impairment as aspect of climate anxiety–the lower the level of conscientiousness, the higher the level of functional impairment.	Confirmed
H3c. Future time perspective predicts the experience of climate change–the higher the level of future time perspective, the higher the level of climate change experience.	Rejected
H4a. Openness predicts behavioral engagement in pro-environmental activity–the higher the level of openness, the higher the level of behavioral engagement.	Confirmed
H4b. Conscientiousness predicts current pro-environmental activity–the higher the level of conscientiousness, the higher the level of current pro-environmental activity.	Rejected
H4c. Present hedonism predicts current pro-environmental activity–the lower the level of present hedonism, the higher the level of current pro-environmental activity.	Rejected
H4d. Present fatalism predicts current pro-environmental activity–the lower the level of present fatalism, the higher the level of current pro-environmental activity.	Rejected
H4e. Future time perspective predicts current pro-environmental activity–the higher the level of future time perspective, the higher the level of current pro-environmental activity.	Confirmed
H5a. Openness predicts planned pro-environmental activity–the higher the level of openness, the higher the level of planned pro-environmental activity.	Confirmed
H5b. Neuroticism predicts planned pro-environmental activity–the higher the level of neuroticism, the higher the level of planned pro-environmental activity.	Confirmed
H5c. Future time perspective predicts planned pro-environmental activity–the higher the level of future time perspective, the higher the level of planned pro-environmental activity.	Rejected

### Strengths of the study

Our findings add to existing knowledge in several ways. The broad selection of variables in the research program is undoubtedly an asset. Novel aspects of the research are the inclusion of not only an index of climate change knowledge, but also belief in climate myths, and examination of their associations with climate anxiety and a range of climate change related emotions (including those not indicative of distress) and actions in the face of the climate crisis. Investigating the links between the three aspects of attitudes toward climate change (cognitive, affective, and behavioral aspects), which have been studied separately, and their personality determinants is also a novel research idea.Our data on the positive but weak correlation between environmental knowledge and PEB is supported by previous research findings [40, 41]. Additionally, the notion that having reliable knowledge about climate change translates into greater awareness of the seriousness of the situation, reflected in the configuration of emotions experienced, its possible consequences, and motivation to take pro-environmental actions is reinforced not only by the negative correlation between the climate myths belief index and PEB, but also by the data supporting the assumption that the knowledge index is weakly negatively correlated with the climate myths belief index. As we have demonstrated empirically, indifference and calmness in the face of the climate crisis are emotional states that co-occur with belief in climate myths. Thus, the results of our study provide arguments in favor of undertaking pro-environmental education and disseminating scientifically verified knowledge on this topic. At the same time, the data we obtained on the intrapsychic determinants of the tendency to adhere to climate myths (whose predictors are the past positive perspective–positive, openness and neuroticism–negative) suggest these variables must be taken into account as individual factors that may limit the effectiveness of the educational process. The possibility of modifying these aspects of personality is limited and–given the known relationship between past positive perspective and well-being [42, 43]–attempts to change this time perspective would be unethical. Hence, it seems necessary not to modify but to balance it. The past positive perspective, which is generally conducive to life satisfaction but is also associated with traditionalism and sentimentality [34], neither of which seems beneficial in the context of the climate crisis, should be balanced by strengthening the future perspective towards an optimal balanced TP [44]. This can be achieved, for example, by following the method described by Zimbardo, Sword, and Sword [45].Considering a wide range of TPs, i.e., retrospective, present, and future orientations, allowed us to fill the gaps in knowledge concerning their relation to PEA. Research linking TP and environmental issues still focuses mainly on the future perspective [35]. As confirmed by our study, future TP affects current PEB, as it was shown to be its predictor (explaining slightly more than 2% of the variance of this variable). It also influences the level of involvement in pro-climate activity, as measured by the CCA scale (explaining 3.05% of the variance of this variable). The future perspective is additionally a predictor of the estimated probability of making changes in the level of pro-environmental activity. It also shows a positive relationship with feelings of mobilization and energy, and a negative relationship with helplessness in the face of crisis. Interestingly, future TP does not affect the strength of the planned PEB index, nor, somewhat surprisingly, does it affect the level of climate anxiety in any of its aspects as measured by the CCA scale. Equally puzzling is the small but statistically significant positive effect of the present fatalistic TP on the level of planned environmental action. Present fatalistic refers to the perception of a lack of personal influence on the situation, which is seen as determined by more or less predetermined external forces (such as fate). It is expressed, in part, by the belief that whatever one does, the outcome is a foregone conclusion. Previous research indicates a negative association between external locus of control and PEA and PEB [23, 46]. The present fatalistic orientation does not encourage current PEB; it only encourages its planning, which can be described as fantasizing about undertaking such activities. However, the relationships captured should be verified in further research.

Experiencing climate anxiety, whose symptoms include cognitive-emotional and functional impairment, appears to be conditioned to some extent by the present hedonistic TP (positive, explaining 2.21% of the variance in the former variable and 2.04% of the latter) and the past positive TP (negative, 2.28% of the variance in the latter). Given the nature of the present hedonistic TP and the findings regarding its relationship with PEB [34, 47], it is understandable that an orientation that is inclined to focus on and seek pleasure, even if short-lived, gives rise to anxiety in the face of a progressive climate crisis. A poorly expressed past positive perspective, which is associated with having resources in the form of positive, empowering past experiences and a strong sense of identity, results in impaired functioning in the course of climate anxiety.

There were also new findings regarding the core domains of personality in the context of experiencing the climate situation and PEB. This represents significant progress in this area, as the findings indicate that examination of the relationship between current and planned PEBs and domain-level personality traits can significantly contribute to the characterization of PEIs, as well as to knowledge of correlates and the sources of climate anxiety. They also shed light on the underlying trait-based drivers of PEB. Experience of the negative effects of climate anxiety that are expressed in cognitive-emotional impairment is influenced by such personality traits as openness-positive, conscientiousness-negative, and neuroticism-positive, while functional impairment is associated with the first two of these core traits. Correlation analysis captured a moderately strong positive relationship between current PEB and the openness trait, as well as a weak correlation with conscientiousness and extraversion. Planned PEB is related to openness and neuroticism. Regression analysis identified the core traits of openness-positive and agreeableness-negative as predictors of current PEBs, while openness-positive and neuroticism-positive domains were predictors of planned PEBs. These results are generally in line with previous findings [7, 24, 48]. Openness conditions intellectual curiosity, willingness to acquire new knowledge, and flexibility of cognitive and behavioral strategies; it is associated with appreciation of beauty, including the natural environment, which helps to understand the empirically demonstrated association of this trait with PEB. Agreeableness is a trait that is associated with empathy and altruistic behavior; altruism is one of the specific traits that comprises the agreeableness dimension (the mid-level facet of Big Five agreeableness) [49]. It seems possible that PEBs may be undertaken because they are perceived as contributing to the good of others and as socially acceptable and preferred (Markowitz et al., 2012) [7]. Reports on the links between neuroticism and PEBs are varied. The model in our study addressed this problem by distinguishing between current and planned actions. This enabled us to study not only intentions motivated by neurotic anxiety, but also actual steps taken and the degree of discrepancy between them. Neuroticism was found to be a predictor of the PEBs that individuals intend to undertake in the future. The links identified between PEBs and various aspects of personality may indicate underlying motives that support and sustain the current and planned actions.

### Limitations of the study

Despite its contribution to the body of knowledge, the study presented here has its limitations. Firstly, the findings were based on a relatively small sample consisting only of Poles, which limits the possibility of generalizing the conclusions. It would be worth repeating the study on a larger and more diverse sample, from other countries and socio-cultural traditions and affected to different degrees by the climate crisis. Due to the elaborate research model and the large number of methods completed by the respondents, we refrained from controlling for some demographic variables, such as the political orientation or income level of the respondents, which could have been related to pro-environmental attitudes and behaviors. It would be worthwhile for subsequent studies to take these into account. Secondly, the results indicate significant but low correlation coefficients and regression coefficients. It is worth keeping in mind that low coefficient values are not exceptional in psychological research and may have practical meaning. However, they may be the result of insufficiently reliable research methods. In some scales, Cronbach’s alpha coefficient was lower than recommended (0.70), which may have been due to the use of abbreviated versions of the methods. Because the research program was extensive and included many indicators at the same time, the methods needed to be as short as possible so as not to overburden the subjects. In addition, the research was conducted using self-report measures, whose validity in relation to PEB is sometimes questioned [50].

## Conclusions

The results mostly verify the basic premises of the study. They indicate that basic personality domains and TPs are significant psychological constructs that should be taken into account in research concerning individual experiences of the climate crisis and engaging in PEB. While our findings confirm and, in some respects, enrich existing knowledge on the correlates and predictors of belief in climate myths, climate emotions, and PEBs, they should be replicated and validated in studies on more diverse groups of individuals.

## Supporting information

S1 Appendix Climate myth beliefs scale. (DOCX)

S2 Appendix Knowledge test. (DOCX)

S3 Appendix Climate emotion scale. (DOCX)

S4 Appendix Inventory of current pro-environmental activities. (DOCX)
