# Supplementary material for: Climate change beliefs, emotions and pro-environmental behaviors among adults: The role of core personality traits and the time perspective
Source: PLoS One. 2024 Apr 10;19(4):e0300246. doi: 10.1371/journal.pone.0300246 (PMC11006203; doi:10.1371/journal.pone.0300246)
Supplement: S1 Appendix — (DOCX) [file pone.0300246.s001.docx]

***Supporting Information: Climate myth belief scale***

| **What are your beliefs about climate change?**  Indicate your opinion about the statements below by assigning them stars – the more stars, the stronger your belief that the statement is true. One star means that you do not agree with this view.   1. The increase in average global temperatures is due to natural causes. 2. The climate has changed before – the changes happening now are part of the natural cycle of cooling and warming that has taken place in the past. 3. People are not significant enough to affect the climate of the entire planet. 4. There is no climate change – it’s just a conspiracy by scientists. 5. Counteracting climate change is nothing urgent. 6. Climate policy is too expensive. Poland can’t afford it. 7. It is unprofitable for Poland to use other energy sources besides coal. 8. The average citizen has no influence on climate policy. 9. Poland doesn’t have to protect the climate, because it is responsible for only 1% of global carbon dioxide emissions. 10. Planting forests is sufficient to reduce greenhouse gas emissions by Poland. |
| --- |
