# Supplementary material for: Climate change beliefs, emotions and pro-environmental behaviors among adults: The role of core personality traits and the time perspective
Source: PLoS One. 2024 Apr 10;19(4):e0300246. doi: 10.1371/journal.pone.0300246 (PMC11006203; doi:10.1371/journal.pone.0300246)
Supplement: S2 Appendix — (DOCX) [file pone.0300246.s002.docx]

***Supporting Information: Knowledge test about climate and its changes***

| Please answer the questions concerning knowledge about climate change.  Each question has only one correct answer.  A natural cause of climate change is:  a. increased concentrations of greenhouse gases  b. changes in the activity of the Sun  c. an increase in air pollution  d. changes in soil composition  The main anthropogenic (dependent on humans) cause of modern climate change is:  a. increased concentrations of greenhouse gases  b. changes in the activity of the Sun  c. changes in the composition of the atmosphere due to volcanic eruptions  d. changes in atmospheric circulation  The greenhouse effect is:  a. a natural phenomenon where the temperature of the planet’s surface is increased by the presence of the atmosphere  b. caused by a lack of atmosphere  c. a natural phenomenon where the temperature of the planet’s surface is decreased by the presence of the atmosphere  d. caused by a deficiency of oxygen  Greenhouse gases include:  a. oxygen  b. carbon dioxide (CO_2_)  c. nitrogen  d. argon  The meteorological element that is most susceptible to climate change is:  a. air temperature  b. precipitation  c. wind  d. air pressure  Which of these international organizations is responsible for regularly preparing climate change reports?  a. United Nations Environment Programme (UNEP)  b. Intergovernmental Panel on Climate Change (IPCC)  c. World Meteorological Organization (WMO)  d. International Council for Science (ICSU)  Which of the below is the main source of methane?  a. mines  b. cattle  c. car exhaust  d. water vapor  At what rate (°C/decade) did the temperature in Poland increase in the last few decades of the 20th century?  a. 0.1°C/decade  b. 0.5°C/decade  c. 0.3°C/decade  d. 0.7°C/decade  When did the air temperature begin to increase significantly in Poland?  a. 1980s  b. 1950s  c. 1930s  d. 1970s  There have always been weather extremes. The extremes currently observed are:  a. rarer and less severe  b. more frequent, stronger, and more troublesome  c. infrequent  d. less common and less troublesome  In recent years, due to climate change, Poland has experienced:  a. a reduction in temperature  b. an increase in temperature and more frequent extreme weather phenomena  c. the absence of drought  d. the absence of hot weather  Despite the absence of distinct trends in precipitation in Poland:  a. the variability of precipitation is increasing  b. precipitation is decreasing  c. there have been no changes in precipitation  d. I don’t know  Renewable energy sources include:  a. coal  b. solar energy  c. petroleum  d. atomic energy  What can contribute most to improving the state of the environment?  a. development of renewable energy  b. limiting travel  c. development of industry  d. nothing, people have no influence on it  We counteract climate change if possible, and if it is not possible, then we:  a. move to another place  b. find ways to adapt  c. do nothing  d. look for positive aspects of the changes |
| --- |
