# Supplementary material for: Climate change beliefs, emotions and pro-environmental behaviors among adults: The role of core personality traits and the time perspective
Source: PLoS One. 2024 Apr 10;19(4):e0300246. doi: 10.1371/journal.pone.0300246 (PMC11006203; doi:10.1371/journal.pone.0300246)
Supplement: S3 Appendix — (DOCX) [file pone.0300246.s003.docx]

***Supporting Information: Climate emotion scale***

| **Thinking about climate change right now makes me feel …**  The more stars you assign to the feeling, the stronger it is. One star means the feeling is minimal or you don’t experience it at all.  **Sad**  **Depressed**  **Angry**  **Powerless**  **Scared**  **Worried**  **Mobilized**  **Indifferent**  **Anxious**  **Helpless**  **Tense**  **Concerned**  **Hopeless**  **Full of energy**    **Calm** |
| --- |
