# Supplementary material for: Climate change beliefs, emotions and pro-environmental behaviors among adults: The role of core personality traits and the time perspective
Source: PLoS One. 2024 Apr 10;19(4):e0300246. doi: 10.1371/journal.pone.0300246 (PMC11006203; doi:10.1371/journal.pone.0300246)
Supplement: S4 Appendix — (DOCX) [file pone.0300246.s004.docx]

***Supporting Information: Inventory of current pro-environmental activities***

| **How do you look after the planet in your everyday life?**  Below are examples of behavior that can mitigate the effects of climate change. Using the scale below, indicate the degree to which you engage in each type of behavior:  1 – not at all  2 – rarely  3 – fairly often  4 – often  5 – always  1. I save water (for example: I take a shower instead of a bath, I turn off the water while brushing my teeth, I run the washing machine when it is full, I don’t water my lawn)   \| 1 2 3 4 5 \| \| --- \|   2. I save electricity (for example: I switch off lights when they aren’t needed, I use energy-saving light bulbs, I use appliances that use as little energy as possible)   \| 1 2 3 4 5 \| \| --- \|   3. I don’t use single-use items (such as cutlery, cups, paper towels, packaging, plastic bags)   \| 1 2 3 4 5 \| \| --- \|   4. When I buy clothes, I make an effort not to harm the planet (I don’t succumb to fashion trends, I have few clothes but of good quality, I like buying clothes made of recycled fiber, I exchange clothes with others or give them to charity)   \| 1 2 3 4 5 \| \| --- \|   5. I reduce the amount of energy needed to heat and cool my home (for example: I seal the windows in winter, I keep the temperature at 18-20 degrees, during hot weather I open the windows when the outdoor temperature goes down instead of using air conditioning)   \| 1 2 3 4 5 \| \| --- \|   6. I try not to eat animal products; I prefer a plant-based diet.   \| 1 2 3 4 5 \| \| --- \|   7. When I shop, I try to harm the environment as little as possible (for example: I buy products sold unpackaged or in paper or glass packaging, but not plastic, I pay attention to labels indicating that the product is environmentally friendly, I buy local products that don’t need to be transported long distances)   \| 1 2 3 4 5 \| \| --- \|   8. I try to use transportation that minimizes my carbon footprint (for example, I use public transportation, I ride my bike, my car is energy-efficient, I limit plane travel)   \| 1 2 3 4 5 \| \| --- \|   9. I try to produce as little trash as possible and dispose of it responsibly (for example: I sort my trash and reduce its volume, I take expired medicines to the pharmacy, I take hazardous waste and large appliances to the appropriate sites or arrange for them to be picked up, I use multi-use products, I compost)   \| 1 2 3 4 5 \| \| --- \|   10. I take pro-environment initiatives (for example, I propose/introduce pro-environment ideas at my school or work, I try to learn as much as possible about environmental activities and campaigns, I join them, I sign petitions, I take part in climate demonstrations)   \| 1 2 3 4 5 \| \| --- \| |
| --- | --- | --- | --- | --- | --- | --- | --- | --- | --- | --- |
